# Variable Cultural Acquisition Costs Constrain Cumulative Cultural Evolution

**DOI:** 10.1371/journal.pone.0018239

**Published:** 2011-03-30

**Authors:** Alex Mesoudi

**Affiliations:** Biological and Experimental Psychology Group, School of Biological and Chemical Sciences, Queen Mary University of London, London, United Kingdom; Durham University, United Kingdom

## Abstract

One of the hallmarks of the human species is our capacity for cumulative culture, in which beneficial knowledge and technology is accumulated over successive generations. Yet previous analyses of cumulative cultural change have failed to consider the possibility that as cultural complexity accumulates, it becomes increasingly costly for each new generation to acquire from the previous generation. In principle this may result in an upper limit on the cultural complexity that can be accumulated, at which point accumulated knowledge is so costly and time-consuming to acquire that further innovation is not possible. In this paper I first review existing empirical analyses of the history of science and technology that support the possibility that cultural acquisition costs may constrain cumulative cultural evolution. I then present macroscopic and individual-based models of cumulative cultural evolution that explore the consequences of this assumption of variable cultural acquisition costs, showing that making acquisition costs vary with cultural complexity causes the latter to reach an upper limit above which no further innovation can occur. These models further explore the consequences of different cultural transmission rules (directly biased, indirectly biased and unbiased transmission), population size, and cultural innovations that themselves reduce innovation or acquisition costs.

## Introduction

One of the hallmarks of human culture is that it is *cumulative*. Beneficial innovations are accumulated and combined over time resulting in knowledge and technology that could not have been invented by a single individual on their own [1], [2]. While many species exhibit culturally-transmitted regional traditions, from chimpanzee tool using traditions [3] to birdsong dialects [4], only humans appear able to accumulate cultural modifications in this way [5]. This cumulative property is of huge significance: it transforms culture into a second evolutionary inheritance system [6]–[8] that is able to generate complex cultural adaptations, from agricultural methods to medical knowledge to mass transportation and communication technologies, that have allowed our species to rapidly and successfully colonise virtually every terrestrial environment on the planet [9], [10]. Despite this significance, little is known about the mechanisms and functions of cumulative cultural evolution. Comparative studies with non-human primates are beginning to delineate the cognitive mechanisms that underpin the capacity for cumulative culture [5], [11]. The present study focuses on the functional aspects of cumulative cultural evolution using analytical and simulation models, taking the capacity for cumulative culture as a given. In particular, I focus here on a previously unexplored constraint on cumulative cultural evolution: the increasing cost of acquiring increasingly complex accumulated knowledge. The following section reviews empirical studies of cumulative culture that provide real-world dynamics against which to compare model output, and empirical support for the claim that cultural acquisition costs are increasing, before presenting macroscopic (Model 1) and individual-based (Model 2) models of constrained cumulative cultural evolution.

### Empirical studies of cumulative culture

A well-documented example of cumulative cultural evolution is seen in the growth of scientific knowledge [12]. Historians of science have detailed how scientific knowledge has gradually accumulated over successive generations of scientists, with each new generation building on the advances of previous generations. To paraphrase Isaac Newton, each new generation of scientists can only see further by “standing on the shoulders of giants”. Mathematics, to which Newton himself contributed, is an illustrative example of how scientific knowledge slowly accumulates over successive generations and thousands of years. Only after Babylonian scholars invented numerical notation and basic arithmetic in around 2000 BC could Greek and Arab scholars subsequently develop geometry and algebra respectively, which then allowed Newton, Liebniz and other Europeans to invent calculus and mechanics in the 17^th^ century, through to present-day mathematics [13], [14].

**Figure 1:**
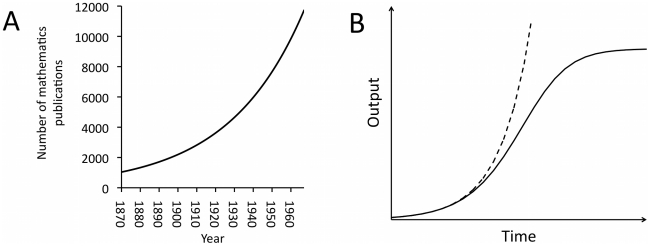
Exponential growth in scientific knowledge. (A) Empirically-derived exponential growth in mathematical knowledge as measured by the number of published abstracts in mathematics from 1868–1965. The curve shown is a best-fit to data reported in May [17], regression equation n = 1400e^0.025(t-1880)^. (B) Price [12] argues that exponential increases in scientific output such as those documented by May (dashed line) are actually the initial part of a logistic growth rate (solid line), eventually reaching a saturation point due to constraints on cumulative cultural evolution.

Quantitative measures of the accumulation of scientific knowledge can be obtained using metrics such as the number of published papers in scientific journals, as well as more direct indices such as the number of planets or species discovered or the number of chemical substances created [12], [15]–[17]. These quantitative “scientometric” analyses have generally revealed an exponential increase in scientific knowledge over time, such as that illustrated in Figure 1A describing the number of published abstracts in mathematics [17]. In a landmark scientometric publication, Price [12] showed that this exponential increase is typical of many scientific fields and that the number of published papers or abstracts generally doubles every 10–15 years. Yet while Price [12] is commonly cited as having shown that scientific knowledge increases exponentially, he also argued that this exponential increase cannot continue indefinitely, and is likely to be the initial part of a logistic growth curve with an eventual upper limit, or saturation point, as shown in Figure 1B (“all the apparently exponential laws of growth must ultimately be logistic”: ref [12], p.30, see in particular Price's Figure 5). Indeed, more recent analyses of scientific accumulation have found evidence of knowledge saturation in certain fields, such as in the discovery of new species or planets [15]. A similar saturation has been observed for research and development in industry [18]: despite increasing expenditure on research and development during the latter part of the 20^th^ century, the number of patents produced per researcher has declined over the same period. These recent trends necessitate a consideration of potential constraints on cumulative cultural evolution.

One potential constraint is that the cost of acquiring the previous generation's accumulated cultural knowledge increases with the magnitude or complexity of that knowledge. It seems reasonable to assume that more-complex knowledge, such as knowledge of quantum physics or the knowledge required to construct computers and space shuttles, takes longer to acquire and has greater scope for copying error than earlier knowledge, such as knowledge of Newtonian physics or the knowledge required to construct stone tools. Indeed, this would seem to be inherent in the very definition of cumulative culture: if beneficial modifications are successively built up over time, then people in later generations will, by definition, have more accumulated knowledge to acquire than people in earlier generations. Assuming that people have a limited, finite amount of time in their lives to devote to acquiring previously accumulated knowledge, there would theoretically come a point at which so much has to be learned that there is no time remaining for innovation, and accumulation will cease.

**Figure 2:**
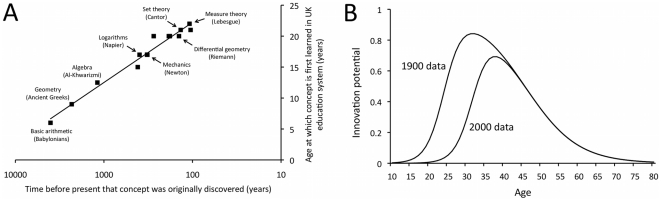
Evidence for increasing cultural acquisition costs. (A) Individual ontogeny recapitulates cultural history for mathematical knowledge: children learn mathematical concepts in the same order that they were first invented historically. The line is a best-fit logarithmic function with R^2^ = 0.97. See Text S1 and Table S1 for sources. (B) Jones' [19] maximum likelihood functions of the probability of a scientist or inventor producing a significant scientific or technological innovation (as measured by the awarding of a Nobel prize or entry in prominent technological almanacs) as a function of the innovator's age, separately for the years 1900 and 2000. Over this 100-year period the peak age of innovation has increased by approximately 6 years, and overall innovation rates have decreased. Functions are derived from equation (3) and Table 2 in ref. [19], recreating that paper's Figure 4.

This prediction rests partly on the assumption that individual learning recapitulates history, in other words, that people learn during their lifetimes a sequence of concepts or skills that have previously been accumulated historically. While this assumption may not apply to all cultural domains, certain domains of scientific knowledge do appear to show this recapitulation. Figure 2A shows how present-day mathematics education during a single lifetime recapitulates the order in which concepts were discovered in human history, from basic counting and arithmetic (invented by Babylonian scholars in approximately 2000 BC and learned at age 5–7 in the UK) to algebra (formulated most extensively by Arab scholars such as Al-Khwarizmi and learned at age 11–14) to calculus and mechanics (invented by Newton and others in the late 1680s and learned at age 16–18) to measure theory (developed at the turn of the 20^th^ century by Lebesgue, and learned at Masters level at a minimum age of 22). Each stage is cumulative: Newtonian mechanics could not have been invented (and cannot be learned) until algebra had been invented (learned), which in turn could not have been invented (learned) without knowledge of basic counting and arithmetic. Figure 2A shows how mathematics appears to be particularly subject to constraints due to increasing complexity: UK Masters-level students do not learn anything that was originally discovered after around 1900. Note, however, that this historical/educational sequence omits suboptimal knowledge that temporarily hindered historical accumulation, such as the Babylonian base-60 system (rather than the currently used base-10 decimal system), which are not learned by present-day schoolchildren. These suboptimal traits are considered in Model 2 below.

Direct evidence for the increasing cost of acquiring previous knowledge and the consequent reduction in innovation is provided in a recent analysis by Jones [19], who found an increase over the last century in the mean age at which Nobel prize winners made their prize-winning contribution, and the mean age at which inventors produced inventions that warrant entry into almanacs of significant technological advances. Figure 2B plots the maximum likelihood functions derived from Jones' [19] analysis of 294 scientists/inventors, showing that the peak probability of producing a significant scientific or technological advance increased from around 32 years of age in 1900 to approximately 38 years of age in 2000. Further analyses showed that this increase is not due to a general increase in lifespan, and can be directly attributed to an increasingly long training period. This can be seen in Figure 2B: while the post-40 decline in productivity is identical for the 1900 and 2000 curves, the age shift occurs at the beginning of life. Indeed, the age at which Nobel prize winners received their PhDs increased by a mean of 4 years over this 100-year period [19]. Note also from Figure 2B the decline in absolute productivity in 2000 compared to 1900, echoing the aforementioned decline in patents per researcher that has occurred concurrently in industry [18]. These empirical phenomena – an increase in the length of training required to make a significant discovery and a decrease in productivity – suggest that increasingly complex knowledge is becoming increasingly more costly and time-consuming to acquire, and is consequently constraining (or may constrain in the future) further accumulation.

Previous analyses of cumulative cultural evolution [20]–[24] have modelled the population-wide increase in some measure of “cultural complexity” as a function of variables such as population size, innovation rate and transmission error. These models have generated valuable insights such as that cumulative culture will be influenced by population size, which can explain the loss of cultural complexity in small populations [20] and gains in cultural complexity as a result of increasing population size [22], or that the exponential increase in cultural complexity reported above for scientific knowledge can be explained in terms of positive feedback between cultural transmission and creativity/innovation [21]. A problematic assumption of these models, however, is that they do not incorporate the aforementioned increasing costs of increasingly-complex accumulated knowledge. The present study explores the consequences of this additional “variable cultural acquisition cost” assumption for cumulative cultural evolution, first by modifying an existing macroscopic, population-based model (Model 1), and then by constructing a more detailed individual-based model (Model 2). The latter individual-based model allows the explicit tracking of individuals and their cultural traits, providing a more direct simulation of cumulative cultural evolution and its constraints.

## Methods

### Model 1 (Macroscopic Model)

Model 1 adds the assumption of variable cultural acquisition costs to a previous model constructed by Henrich [20]. In this model, a population is assumed to comprise *N* individuals each of whom has some level of culturally transmitted skill denoted *z_i_* (where subscript *i* identifies each individual, with *i* = 1, 2…*N*). This culturally transmitted skill might be the complexity of the toolkit that the individual is able to manufacture or use, or the complexity of the scientific or ecological knowledge that the individual possesses. Each member of each new generation of *N* individuals acquires their *z_i_* value from the member of the previous generation who has the highest *z* value, *z_h_* (i.e. directly biased transmission: [8]). This transmission is inaccurate, reflecting real-life difficulties of inference and communication [25], [26]. The naïve individual's *z_i_* value is drawn from a Gumbel distribution with mode *z_h_* - *α* and dispersion *β*. The parameter *α* represents systematic transmission error that degrades the skill, while *β* represents unsystematic noise in transmission that occasionally may result in an improved skill level relative to *z_h_*. Using the Price equation [27], Henrich [20] showed that the between-generation change in mean *z* value across in the entire population, 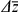, is given by:
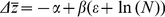
(1)where *ε* is the Euler-Gamma constant (*ε*≈0.577). When this change is positive (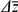
>0) then culture is said to accumulate, which occurs when systematic transmission error *α* is relatively low, when random inference *β* is relatively high, and population size *N* is relatively large.

In order to add the assumption that increasingly complex cultural knowledge is more costly to acquire, Eq. 1 can be modified to make the error parameter *α* a linear function of the previous generation's accumulated skill level 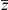
_t-1_. This error term *α* now captures both errors in transmission and the cost of acquiring previous knowledge, both of which would increase with the amount of knowledge that must be acquired (represented by 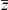
_t-1_). The recursion therefore becomes:
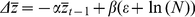
(2)where 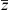
_t-1_ gives the accumulated cultural complexity of the previous generation.

### Model 2 (Individual-Based Model)

While macroscopic models such as Model 1 are useful first approximations of the dynamics of cumulative cultural evolution, such models have several limitations. First, conceptualising “culture” as a single continuous variable (e.g. *z*) does not reflect the discrete basis of technological and scientific change. Historians of science and technology typically view cultural change as the invention and accumulation of discrete innovations, such as James Watt's modification of the existing steam engine by adding a separate condensation chamber [28], the invention of retractable landing gear in aircraft [29] or the invention of the mathematical concepts and techniques shown in Figure 2A [13], [14]. While the net result of these innovations might be a continuous increase in engine efficiency, aircraft speed etc., at the micro-level this increase is step-wise and the result of discrete contributions by specific individuals. Second, and more importantly, macroscopic models do not typically treat cultural traits as functionally and historically dependent, where each new innovation is dependent on a series of previous functionally-linked modifications. James Watt did not re-invent the steam engine from scratch, he made a minor modification to the existing Newcomen steam engine; algebra could only have been invented once the basic number system was in place, and so on. Indeed, this functional and historical dependence would seem to be the defining characteristic of cumulative cultural evolution. The modification made to Henrich's [20] model above represents only a crude way of implementing this dependence.

To investigate these more realistic properties of cumulative culture, an individual-based model (Model 2) was constructed to keep track of each individual's knowledge and each discrete trait in the population, and explicitly incorporate inter-individual cultural transmission. Such methods have been used previously to model the change in distribution of cultural traits such as first names and pottery designs over time in response to drift-like random copying and frequency-dependent cultural transmission biases [30], [31]. An individual-based model of cumulative culture by Strimling et al. [32] represents a valuable first attempt to understand the microevolutionary basis of this phenomenon, but does not feature the key assumption of functionally dependent cultural traits that are costly to acquire, nor does it produce an initial exponential increase in knowledge.

Model 2 was implemented in C++ (code available from the author upon request). A population of *N* individuals (indexed by *i*, where *i* = 1,2,3…*N*) engage in cultural transmission and innovation for *T* generations (*t* = 0,1,2…*T*). Each individual learns a set of cultural traits that are functionally sequential, such that earlier traits must be learned before later traits in the sequence can be acquired. Each trait is denoted by *x_s_*, where *x* is an integer from 1 to *X* (in the simulations that follow, *X* is fixed at 100). These integers can be seen as different technological modifications or scientific ideas. The subscript *s* is an integer (where *s* = 1,2,3…∞) indicating the functional level of that trait, with one trait per *s*-level. So an individual with *x_1_* = 42, *x_2_* = 71 and *x_3_* = 13 has acquired three traits, the first labelled 42, the second 71 and the third 13. The *x_3_* trait cannot be learned unless the *x_2_* trait has already been learned, which in turn can only be learned by individuals already knowledgeable of *x_1_*.

In order to track cumulative increases in complexity in the population, each trait *x_s_* is assigned a “trait fitness” of *z_x_* which describes that trait's effectiveness (in the case of technological inventions) or veracity (in the case of scientific theories). Note that these are cultural measures of fitness rather than genetic measures; we are concerned here with changes in cultural traits over time rather than genes, and it is assumed that cultural traits have no bearing on survival or reproduction of individuals. The fitness of each trait within a single *s*-level is drawn independently from an exponential distribution as in Rendell et al. [33], such that there are always a small number of very effective modifications and a large number of minimally effective or neutral modifications. Following [33], the values drawn from the exponential distribution with rate parameter equal to 1 are squared, doubled and rounded to integers. Roughly half of these values have zero fitness, representing non-viable attempts at a solution, with a small number of highly effective traits with fitness around 50. This assumption of multiple, difficult-to-find solutions that vary in their effectiveness is likely to apply to many real-life technological or scientific domains [34].

Trait fitness (*z_x_*) is distinguished from “individual fitness”, *Z_i_*, which is the sum of the fitnesses of all learned traits known by an individual *i* at every *s*-level up to the highest one learned, *s_max_*:
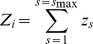
(3)


For example, the three traits listed above, *x_1_*, *x_2_* and *x_3_*, might have trait fitnesses of *z_1_* = 24, *z_2_* = 9 and *z_3_* = 48 respectively. The individual who has learned these three traits (and only these three traits, such that their *s_max_* = 3) therefore has an individual fitness of *Z_i_* = 81. The mean cultural complexity of a population at time *t* is denoted 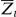 and calculated as the mean of all *N* individuals' *Z_i_* values, allowing a comparison with the equivalent measure of mean cultural complexity, 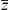 *_t_*, used in Model 1.

During each generation the population is replaced with *N* naive, unknowledgeable individuals. Note that there is no differential reproduction of individuals given that we are interested in cultural rather than genetic change. Each individual has an “effort budget” of *λ*, which represents the total amount of effort that an individual can devote in their lifetime to learning cultural traits (either individually or socially), representing the constraint on accumulation introduced in the macroscopic model above. Every individual of the new generation goes through an initial stage of copying from the previous generation (i.e. oblique cultural transmission: [35]). Three alternative copying rules were implemented: directly biased, indirectly biased and unbiased transmission [8]. For indirect bias, new individuals copy all of the traits exhibited by the single individual in the previous generation who has the highest individual fitness, *Z_i_*. For direct bias, new individuals go through each *s*-level achieved by at least one member of the previous generation and copy the trait with the highest trait fitness, *z_x_*. Indirect bias involves copying successful individuals, whereas direct bias involves copying effective traits. Both are plausible copying rules [8]: copying successful individuals is a quick and cheap way of acquiring effective behaviour but may involve the acquisition of some suboptimal traits, whereas direct bias is more likely to result in the acquisition of the most effective traits but with greater time and cost (although these costs were not directly simulated in the present model). This distinction cannot be easily explored in the macroscopic model of Henrich [20], nor in Model 1 above, because individuals and traits are not explicitly tracked. Finally, for unbiased transmission, new individuals copy all of the traits of a randomly-selected member of the previous generation. While probably unrealistic with respect to real-life scientific and technological change, this provides a baseline for assessing the effectiveness of the two non-random biases.

Copying, whether directly biased, indirectly biased or unbiased, incurs a cost *c_s_* per trait, where *c_s_* is measured in effort units. This cost is fixed for every trait irrespective of the fitness of the trait, so a trait of fitness *z* = 50 is just as costly to acquire as a trait of fitness *z* = 5. This is a simplifying assumption based on the intuition that there does not seem to be any systematic correlation between ease of learning and trait fitness. In some cases suboptimal traits, such as the base-60 system or Roman numerals, appear to be more difficult to learn than higher-fitness traits such as the base-10 system or Arabic numerals. In other cases optimal traits, such as Darwinian evolutionary theory, appear more difficult to learn than suboptimal traits, such as teleological or Lamarckian evolutionary theories [36], [37]. In the absence of any systematic or empirically determined correlation, a fixed and fitness-independent cost was employed.

Copying proceeds until the learner either (i) learns all of the traits available from the previous generation (either from the most successful individual in the case of indirect bias, collated across all individuals in the case of direct bias, or from a randomly selected individual in the case of unbiased transmission) or (ii) runs out of effort budget. If the learner has any effort budget remaining after copying then innovation occurs. During innovation, the learner randomly selects one of the cultural traits (from 1 to *X*) at the first *s*-level at which no trait has yet been acquired by that individual. If the trait selected is viable (i.e. its associated fitness is greater than zero) then the individual successfully learns that trait, otherwise another trait is chosen at that *s*-level. The assumption that traits with fitness of zero are not learned and thus not accumulated is intended to capture the cumulative aspect of culture, where only effective traits are built upon.

Innovation, whether successful or unsuccessful, incurs a cost *c_i_* per trait, where *c_i_* is measured in effort units. This is again a simplifying assumption, but is intended to reflect the notion that inventors/scientists do not possess the foresight to know in advance which innovations will be most effective [38], and so may expend effort and time on ultimately fruitless lines of research. Innovation proceeds for every successive *s*-level until the learner runs out of effort budget. It is assumed that the cost of innovation is higher than the cost of copying (*c_i_>c_s_*), as is standard in cultural evolution models and reflecting the intuition that it is easier to learn something from someone else than invent it from scratch. The first generation undergoes innovation but not copying.

Finally, in order to explore the positive consequences of increasing complexity, and in particular to explore the reasons for exponential growth in cultural complexity discussed above, it is assumed that both *c_i_* and *c_s_* may also decrease in proportion to the mean level of cultural complexity in the population, 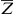 *_t_*. The proportionality constants *µ_i_* and *µ_s_* determine the rate at which these costs decrease. Each individual of generation *t* therefore has a modified *c_i_* of 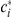 =  (*c_i_* - *µ_i_* 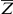
_t-1_) and a modified *c_s_* of 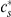 =  (*c_s_* - *µ_s_* 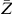
_t-1_), with the constraints 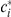
≥1 and 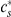
≥1 (otherwise learning becomes costless and accumulation continues to infinity). The assumption that the cost of innovation, *c_i_,* decreases with cultural complexity reflects the idea that certain innovations, such as new instruments, methods or techniques, can increase the likelihood of making further advances. For example, Schummer [16] showed that the exponential growth in the number of chemical substances created by chemists over the last 200 years has been driven endogenously in this manner, such as when a new substance is created using a particular reaction mechanism and this reaction mechanism is then applied to other substance classes to create further substances, rather than as a result of exogenous factors such as an increase in the number of active chemists (i.e. *N* in this model) or funding expenditure. The assumption that the cost of copying, *c_s_*, decreases with cultural complexity is intended to reflect the invention of new means of communication such as the printing press or the internet which make it easier to acquire beneficial cultural traits from the previous generation. This assumption is less empirically-supportable, and these changes may be more appropriately seen as exogenous rather than endogenous (e.g. there is no sense in which the printing press resulted from mathematical knowledge of the 15^th^ century). However, rather than introducing a novel exogenous process, this is examined here endogenously in a parallel manner to the reduction in innovation costs.

## Results

### Model 1 (Macroscopic Model)

**Figure 3:**
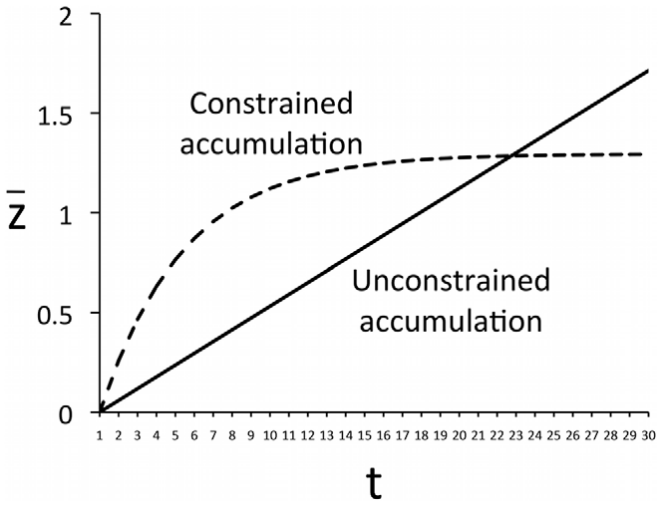
Cultural accumulation over successive generations in Henrich's [**20**] original unconstrained model (Eq. 1) and in the constrained Model 1 (Eq. 2). Parameters: *N* = 100, *α* = 0.2, *β* = 0.05.



Fig. 3 shows the cultural accumulation over time of the original Henrich [20] model as well as the modified Model 1 in which cultural accumulation is constrained by complexity-dependent acquisition costs. While the original model exhibits a linear increase in cultural complexity continuing to infinity, the modified model reaches a stable equilibrium value of cultural complexity of *z_max_*. Setting 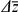 = 0 and rearranging Eq. 1 gives this equilibrium value as :

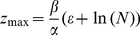
(4)


This simple addition to Henrich's [20] model, one that appears quite plausible on the logical and empirical grounds discussed above, predicts that cumulative cultural evolution should reach some stable upper limit as the knowledge accumulated becomes too complex to successfully acquire and build upon in each generation. It does not, however, generate the initial exponential increase in knowledge shown above to be typical of real-life cultural change.

### Model 2 (Individual-Based Model)

**Figure 4:**
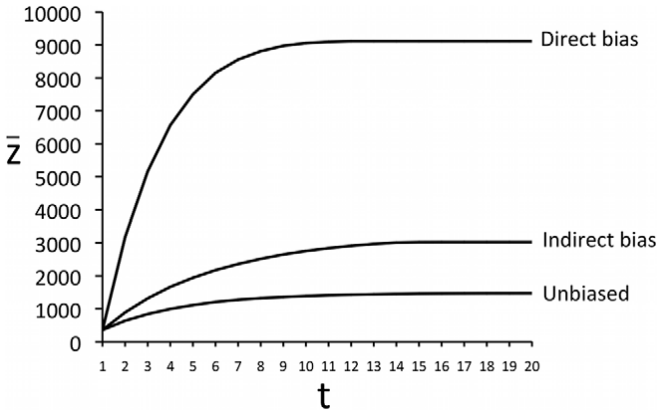
Time series of mean cultural complexity over time in Model 2 indicating that complexity reaches a maximum equilibrium 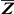 *_max_*. Three alternative copying rules are shown: unbiased transmission (new individuals copy the cultural traits of a randomly selected member of the previous generation), indirectly biased transmission (new individuals copy the cultural traits of the member of the previous generation who had the highest individual fitness) and directly biased transmission (new individuals copy the cultural traits with the highest trait fitnesses across the entire previous generation). Parameters: N = 100, *c_i_* = 10, *c_s_* = 5, *λ* = 1000, *µ_i_* = *µ_s_* = 0; all results are the average of 100 independent runs.

The constraints on learning inherent in the individual-based Model 2 imposed via the *λ*, *c_i_* and *c_s_* terms generated upper limits (denoted 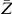 *_max_* as above) on the amount of cultural complexity that can be attained (Figure 4), similar to those observed in the modified macroscopic model above (Figure 3). As shown in Figure 4, the copying rule affects the magnitude of the maximum cultural complexity that is attained, with direct bias resulting in higher 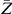 *_max_* than indirect bias, which in turn results in higher 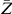 *_max_* than unbiased transmission. This is because direct bias involves the selection of traits of the highest fitness at every *s*-level separately, whereas indirect bias allows suboptimal traits to accumulate when they are exhibited by individuals who nevertheless have the highest overall individual fitness. This hitch-hiking effect is explored further below.

**Figure 5:**
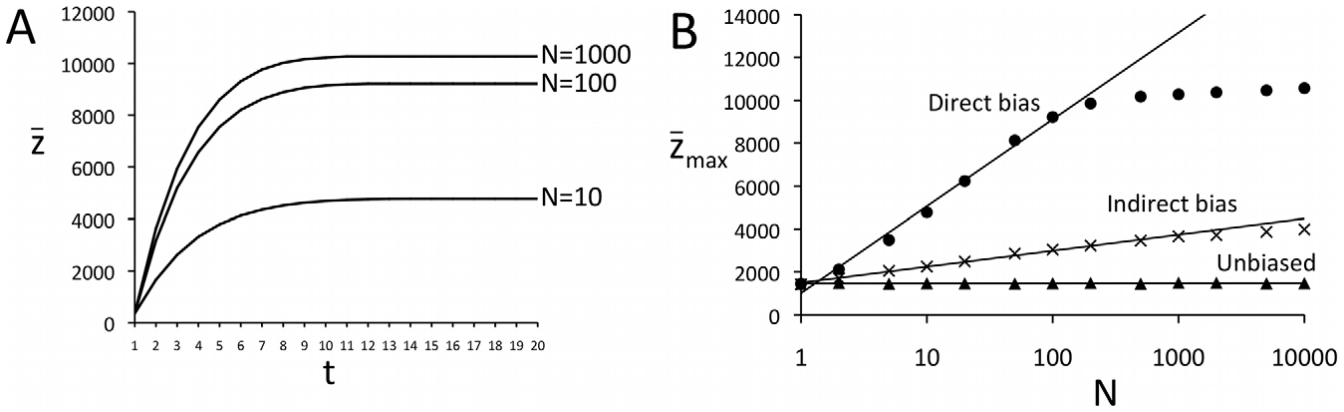
Interaction between population size and cultural complexity in Model 2. (A) Time series of mean cultural complexity over time in Model 2 at different population sizes, *N*, under the assumption of direct bias. (B) Relationship between maximum cultural complexity 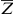 *_max_* and *N* for direct bias, indirect bias and unbiased transmission, and with *N* plotted on a logarithmic scale. For direct bias, maximum cultural complexity 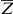 *_max_* increases logarithmically with *N* up to *N* = 100 (line is a logarithmic best-fit with R^2^ = 0.991 for *N*≤100), after which it plateaus. For indirect bias, a similar logarithmic increase followed by a plateau occurs to that under direct bias, but the values of 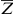 *_max_* are lower and the plateau occurs at higher values of *N* (around *N* = 1000; line is a logarithmic best-fit with R^2^ = 0.994 for *N*≤1000). For unbiased transmission, *N* has no effect on 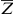 *_max_*. Parameters: *c_i_* = 10, *c_s_* = 5, *λ* = 1000, *µ_i_* = *µ_s_* = 0, all results are the average of 100 independent runs.

Maximum complexity 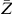 *_max_* also increases with population size, *N* (Figure 5A), replicating Henrich's [20] finding that larger populations can support greater cultural complexity. More individuals in the population mean that rare high-fitness cultural traits are more likely to be discovered during innovation, resulting in higher complexity. Figure 5B shows, for direct bias, a logarithmic relationship between N and 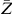 *_max_* until the latter plateaus at around *N* = 100 for these parameter values. This indicates that adding more individuals to the population has the greatest effect at relatively low values of N. At relatively high values of N, adding more individuals has little effect because all high-fitness traits have already been discovered. The point at which further increases in *N* have no effect is roughly equal to *X*, the number of alternative trait values at each *s*-level. When *N*≥*X* then it is likely that at least one individual will discover the optimal trait at that level, and further increases in *N* have little effect. As *X* = 100 here, the point at which this occurs in Figure 5B is when *N*≥100. Figure 5B shows that a similar logarithmic relationship plus plateau also occurs for indirect bias, but it takes larger populations (around *N* = 1000 in this case) to reach the point at which a further increase in *N* has no effect on complexity. *N* has no effect when transmission is unbiased, because a randomly-selected member of the previous generation is no more likely to exhibit traits with high fitness when populations are large compared to when they are small.

**Figure 6:**
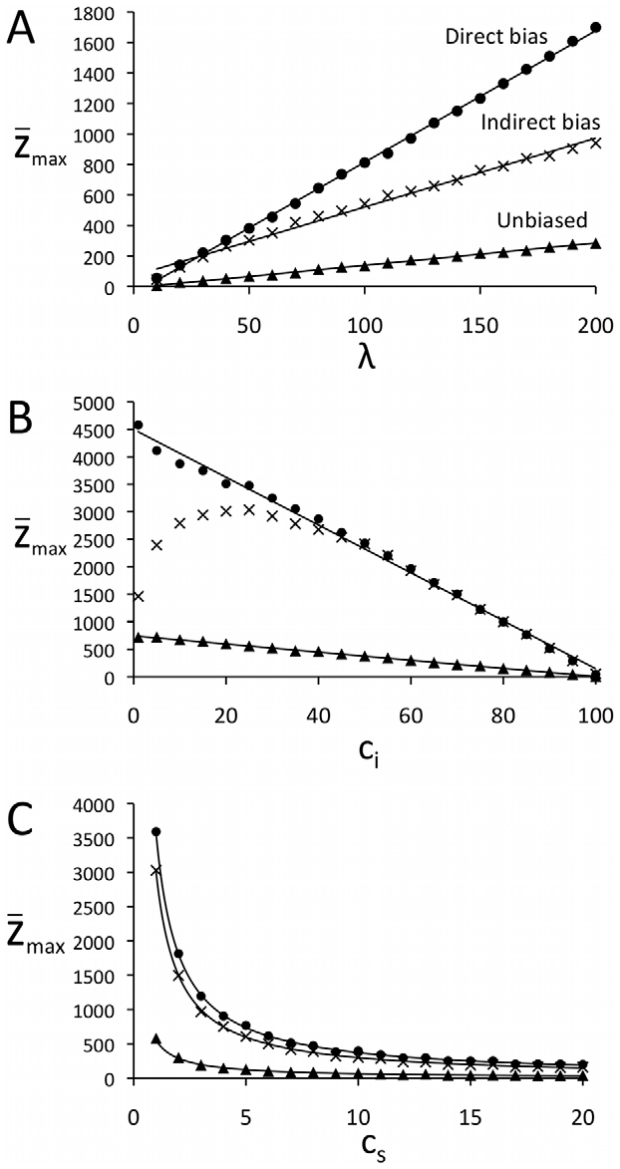
The effect on maximum cultural complexity 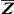 *_max_* of varying (A) lifetime effort budget *λ* (with constant *c_i_* = 10, *c_s_* = 5), (B) innovation cost *c_i_* (with constant *c_s_* = 1, *λ* = 100), and (C) copying cost *c_s_* (with constant *c_i_* = 10, *λ* = 100). Circles indicate direct bias, crosses indicate indirect bias and triangles indicate unbiased transmission. Lines show best-fit functions, in (A) showing a positive linear relationship between 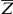
_max_ and *λ* for direct bias (R^2^ = 0.999), indirect bias (R^2^ = 0.991) and unbiased transmission (R^2^ = 0.998); in (B) showing a negative linear relationship between 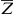 *_max_* and *c_i_* for direct bias (R^2^ = 0.995) and unbiased transmission (R^2^ = 0.999), but for indirect bias only for larger *c_i_* values (for *c_i_*>30, R^2^ = 0.990), with lower *c_i_* values generating lower 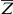 *_max_* values than expected (no best-fit line drawn); and in (C) showing an inverse power law relationship between 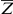 *_max_* and *c_s_* for direct bias (R^2^ = 0.997), indirect bias (R^2^ = 0.997) and unbiased transmission (R^2^ = 0.992). Other parameters: *N* = 100, *X* = 100, *µ_i_* = *µ_s_* = 0, all results are the average of 100 independent runs.



The maximum cultural complexity attainable, 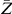 *_max_*, should also depend on the relative magnitudes of *c_i_*, *c_s_* and *λ*, given that lower costs relative to the total effort budget should allow individuals to learn more cultural traits. Figure 6 shows the relationships between 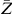 *_max_* and these three variables, separately for directly, indirectly and unbiased transmission. To understand these relationships, we can assume that 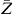 *_max_* correlates with the maximum number of traits that can be accumulated, *s*_max_. It is shown in Text S2 that

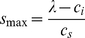
(5)


Equation 5 says that *s_max_*, and by extension 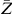 *_max_*, should show a positive linear relationship with *λ*, a negative linear relationship with *c_i_* and an inverse power relationship with *c_s_*. Figure 6A shows that the first prediction for *λ* is upheld for all three transmission rules. Figure 6B shows that *c_i_* exhibits a negative linear relationship with 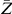 *_max_* as predicted for direct bias and unbiased, but not for indirect bias where low values of *c_i_* produce lower 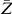 *_max_* than expected. Figure 6C shows that *c_s_* fits the expected inverse power relationship for all three transmission rules.

The initial increase in 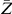 *_max_* at low values of *c_i_* under indirect bias shown in Figure 6B appears counterintuitive: why does increasing the cost of innovation initially cause an increase in the maximum cultural complexity attained? Time-step analyses indicated that this was because indirectly biased cultural transmission with low innovation costs allows suboptimal cultural traits to accumulate. Indirectly biased transmission means that the individual with the highest mean complexity score is copied by the next generation. When *c_i_* is low then a single individual can discover several traits during the innovation stage. If this best individual has acquired several traits at once, then although some of those traits will be of high complexity (given that the individual has the highest mean fitness in the population), some may be of low complexity. When the subsequent generation copies all traits of the best *t*-1 individual, this may include several of these low fitness traits. Given that traits are functionally dependent, earlier low fitness traits cannot be improved upon once they become accumulated, and thus reduce the eventual maximum accumulated cultural complexity. As *c_i_* increases, individuals can acquire fewer traits in a their lifetime, and high fitness individuals are less likely to also have (and transmit) low-fitness traits. This hitch-hiking effect also explains why indirect bias results in lower maximum cultural complexity than direct bias, as shown in Figure 4.

**Figure 7:**
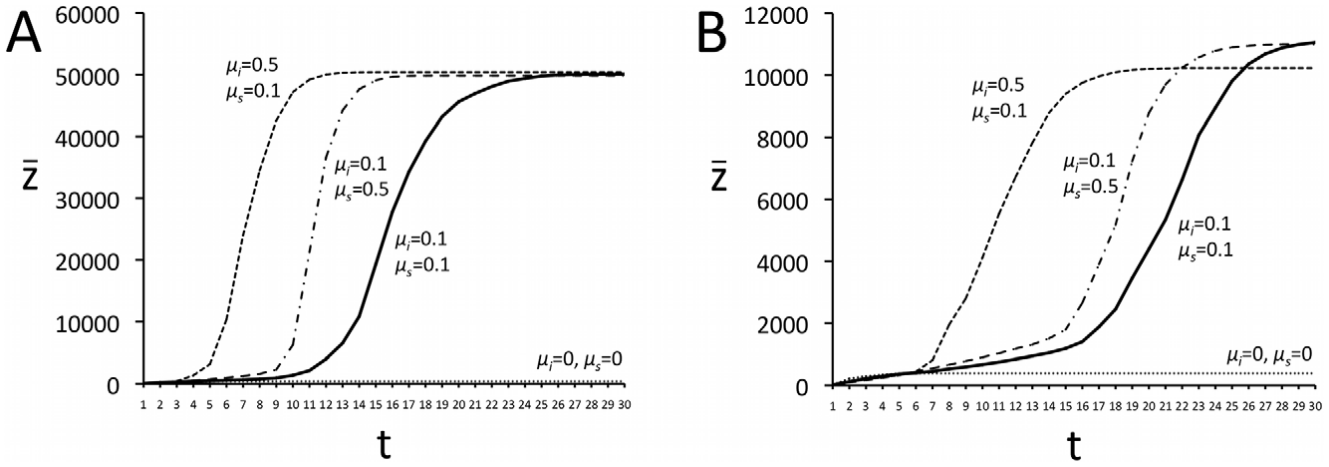
Time series of mean cultural complexity 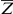 when *c_i_* and *c_s_* decrease in proportion to 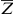 according to proportionality constants *µ_i_* and *µ_s_* respectively. In (A) transmission is directly biased, in (B) transmission is indirectly biased. Other parameters: *N* = 100, *c_i_* = 200, *c_s_* = 100, *λ* = 1000, all results are the average of 100 independent runs.

Finally, consider the case where the cost of innovation *c_i_* and the cost of cultural transmission *c_s_* both decrease in proportion to the mean cultural complexity of the previous generation (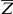 *_t-1_*) according to proportionality constants *µ_i_* and *µ_s_* respectively. When *µ_i_* and *µ_s_* are sufficiently large, then cultural complexity can increase to high values even at normally prohibitively high values of *c_i_* and *c_s_*, as shown in Figure 7 for both direct (Figure 7A) and indirect (Figure 7B) bias (unbiased transmission failed to produce take-offs). In both cases, cultural complexity shows a gradual initial increase as the costs of learning slowly fall, before increasing rapidly and then reaching the constraint imposed by the requirement that 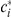
≥1 and 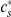
≥1. These curves resemble the logarithmic curve shown in Figure 1B that is argued to represent real-life technological and scientific accumulation. From Figure 7 it can be seen that *µ_i_* and *µ_s_* do not behave in exactly the same way. First, increasing *µ_i_* causes cultural complexity to take off earlier than the same increase in *µ_s_*. Second, whereas for direct bias (Figure 7A) the magnitude of *µ_i_* and *µ_s_* do not affect the final maximum complexity 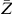 *_max_*, for indirect bias (Figure 7B) a relatively large *µ_i_* reduces 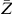 *_max_*, compared to the same increase in *µ_s_*. This is because of the aforementioned accumulation of suboptimal traits that is permitted by indirect bias (see Figure 6B): if *µ_i_* is large then *c_i_* will decrease quickly, and a single individual will be able to acquire multiple traits some of which will be suboptimal.

## Discussion

The aim of this study was to explore a potentially important constraint on cumulative cultural evolution: the increasing costs of acquiring increasingly complex accumulated knowledge. It was suggested that as more, and more complex, knowledge is accumulated over successive generations, it will become increasingly time-consuming and difficult for each new generation to successfully acquire this accumulated knowledge. In principal, there may come a point at which acquisition is so costly that no time is left for innovation, and accumulation ceases. Evidence was reviewed from quantitative analyses of the history of science and technology to support the potential existence of such increasing acquisition costs: first, the acquisition of accumulated knowledge by an individual was shown to recapitulate the historical accumulation of that knowledge, such that later generations have more to acquire than earlier generations; second, while scientific and technological knowledge is known to have increased exponentially over time, certain domains have shown recent slowdowns in the rate of innovation; and third, the mean age at which scientists and inventors make significant contributions to their fields has increased over the last century and this increase can be directly attributed to a longer training period, supporting the assumption that acquisition of prior knowledge is becoming increasingly difficult and time-consuming. Two theoretical models were then presented that explored the consequences of increasing cultural acquisition costs on cumulative cultural evolution. Model 1 extended an existing macroscopic model of cumulative cultural evolution, finding as expected that making acquisition costs dependent on mean population-wide cultural complexity created upper limits on the amount of cultural complexity that can be attained. Model 2 explored this further using individual-based methods in which discrete traits, individuals and transmission paths are explicitly simulated. Here, assuming that individuals have a finite lifetime “effort budget” to devote to either acquiring prior knowledge or innovating new knowledge again produced upper limits on the maximum cultural complexity that can be attained, at which point the entire effort budget is spent acquiring prior knowledge with none left over for further innovation.

The magnitude of this upper limit was shown in Model 2 to depend on several parameters, each of which has potential implications for the study of real-life cumulative cultural evolution. First, as in previous models [20], [22], larger populations supported higher maximum cultural complexity. However, this only occurred up to the limit set by the acquisition costs, above which further increases in population size had no effect (i.e. the plateau in Figure 5B). This is because lifetime learning capacity is an individual characteristic, and is (in this model) unaffected by the number of other individuals in the population. Excessive cultural acquisition costs may therefore constitute a limiting factor on cultural complexity operating independently of demographic constraints. The observation that the number of new patents issued has exhibited a constant or even declining growth rate during the latter part of the 20^th^ century despite an exponential increase in the number of active researchers [18] may constitute evidence of this independence, and warrants further investigation.

Second, directly biased cultural transmission, where individuals copy the most effective cultural traits exhibited across the entire previous generation, allowed higher cultural complexity to accumulate than indirectly biased cultural transmission, where individuals copy all of the traits of the single individual from the previous generation who has the highest total knowledge. This in turn permitted higher complexity than unbiased transmission, where individuals copy all of the traits of a single randomly-chosen individual. In general, while unbiased transmission (i.e. random copying) might explain changes in the distribution of certain cultural traits [30], cumulative cultural evolution requires direct or indirect bias. The disadvantage of indirect bias over direct bias is that indirect bias allows suboptimal traits to accumulate when exhibited by the most-knowledgeable individual. Somewhat counter-intuitively, this was most likely to occur at low innovation costs (see Figure 6B). This is because low innovation costs allow individuals to invent several traits in a single lifetime; while the most knowledgeable individual will have mostly high-fitness traits, some may be suboptimal, and under the assumption of indirect bias these suboptimal traits are copied by subsequent generations along with the optimal traits. High innovation costs mean that individuals can only invent a few or a single trait in their lifetimes, maintaining a closer correlation between trait and individual fitness. With respect to real-life scientific and technological change, we might ask whether past or present educational and apprenticeship systems better resemble either direct or indirect bias. Newton's studies of alchemy or Darwin's theory of pangenesis might represent suboptimal traits potentially copied by subsequent generations via indirect bias due to the overall success of their progenitors. If the primary mode of cultural transmission was indirect bias, then we might predict that such suboptimal traits may have hindered scientific progress more than commonly assumed, as subsequent generations wasted effort in acquiring these traits and devoted less time to innovation. It is also possible that the scientific method contains formal mechanisms (e.g. anonymous peer review) for avoiding the accumulation of suboptimal traits via indirect bias. On the other hand, the awarding of honours (e.g. Nobel prizes) to single individuals may encourage a prestige-based form of indirect bias. Experimental laboratory studies of cultural transmission have shown that people willingly employ indirect bias in preference to individual learning, and that neutral traits can consequently be copied along with functional traits when both are exhibited by successful models [39], [40]. Further experiments might test more systematically the conditions under which people engage in direct and indirect bias when acquiring knowledge and skills accumulated by previous (cultural) generations of participants in the lab, such as in response to the aforementioned innovation costs. Further models and experiments may also add the assumption that direct bias is intrinsically more costly than indirect bias, because it requires surveying the entire population on a trait-by-trait basis rather than copying the single most-successful individual, or that suboptimal traits can be returned to by later generations and improved.

Third, maximum cultural complexity increased with lifetime effort budget, and decreased with increasing costs of both innovation and cultural acquisition (Figure 6). Although such quantities are somewhat abstract, recent scientometric analyses have shown that they may be measured with some degree of accuracy, such as Jones' [19] quantification of an individual's lifetime innovation potential, and the decrease in this potential over the last century due to longer training periods (see Figure 2B). The length of training period, for example, might be seen as equivalent to *c_s_* in Model 2 and measured in time. Further scientometric studies for different domains and over longer time periods might yield parameter estimates that can be used to predict the specific course of future cultural change, and perhaps even estimate when the upper limits observed in the present model might be reached.

Finally, adding the assumption that innovation costs and acquisition costs may both decrease with accumulated complexity generated an initial exponential increase in cultural complexity (Figure 7) that resembles real-life patterns of scientific and technological change [17], [21]. However, the eventual upper limit remained due to the assumption that learning always comprises some cost. The first assumption that innovation costs decrease with complexity was intended to capture the notion that certain innovations, such as new techniques or instruments, make further discoveries more likely. There is evidence from the history of science that new techniques may increase the likelihood of further innovation in this way [16]. The second assumption that acquisition costs also decrease with complexity was intended to represent innovations such as formal education systems, the printing press or the internet, that reduce the cost of cultural acquisition. This assumption is less empirically supportable, and such phenomena may be more appropriately modelled in the future as exogenous. Nevertheless, the S-shaped curves shown in Figure 7 resemble the logistic growth pattern proposed by Price [12] to describe real-life scientific and technological change (Figure 1B), and show that such patterns can be obtained from a set of standard yet minimal assumptions about transmission dynamics and trait distributions developed by cultural evolution researchers [8], [35]. Further historical studies might test the additional prediction that innovations which reduce the cost of further innovation (i.e. *µ_i_*) will generate faster and earlier exponential increases in complexity than innovations that reduce the cost of acquisition (i.e. *µ_s_*).

A limitation of the present models is their unilinearity, with only a single sequential lineage of cultural traits. In reality, cultural evolution is multilinear [41], [42], with several concurrent lines of scientific investigation or technological lineages existing simultaneously. Indeed, a major source of innovation is likely to be the recombination of traits from different lineages [43], which has been suggested to result in an exponential increase in cultural complexity [44]. This was demonstrated analytically by Enquist et al. [21], although that model did not feature the constraints introduced here. Future models might explicitly simulate multiple cultural lineages and within-generation, cross-lineage recombination, in addition to the constraints explored here, in order to provide a better theoretical foundation for cumulative cultural evolution and to guide future historical and experimental studies of this phenomenon.

## Supporting Information

Text S1 **Data sources for** **Figure 2A** **/Table S1.**
(DOC)

Text S2 **Derivation of** **Equation 5** **.**
(DOC)

Table S1 **Exact dates and ages plotted in** **Figure 2A** **.**
(DOC)

## References

[1] Tomasello M (1999). The cultural origins of human cognition..

[2] Boyd R, Richerson PJ (1996). Why culture is common, but cultural evolution is rare.. Proceedings of The British Academy.

[3] Whiten A, Goodall J, McGrew WC, Nishida T, Reynolds V (1999). Cultures in chimpanzees.. Nature.

[4] Catchpole CK, Slater PJB (1995). Bird song: Biological themes and variations..

[5] Tennie C, Call J, Tomasello M (2009). Ratcheting up the ratchet: On the evolution of cumulative culture.. Philosophical Transactions of the Royal Society B.

[6] Mesoudi A, Whiten A, Laland KN (2004). Is human cultural evolution Darwinian? Evidence reviewed from the perspective of The Origin of Species.. Evolution.

[7] Mesoudi A (in press). Cultural evolution..

[8] Boyd R, Richerson PJ (1985). Culture and the evolutionary process..

[9] Richerson PJ, Boyd R (2005). Not by genes alone..

[10] Hill K, Barton M, Hurtado AM (2009). The emergence of human uniqueness.. Evolutionary Anthropology.

[11] Whiten A, McGuigan N, Marshall-Pescini S, Hopper LM (2009). Emulation, imitation, over-imitation and the scope of culture for child and chimpanzee.. Philosophical Transactions of the Royal Society B.

[12] Price DJS (1963). Little science, big science..

[13] Wilder RL (1968). Evolution of mathematical concepts..

[14] Gittleman A (1975). History of mathematics..

[15] Arbesman S (2010). Quantifying the ease of scientific discovery.. Scientometrics.

[16] Schummer J (1997). Scientometric studies on chemistry I: The exponential growth of chemical substances, 1800-1995.. Scientometrics.

[17] May KO (1966). Quantitative growth of the mathematical literature.. Science.

[18] Segerstrom PS (1998). Endogenous growth without scale effects.. American Economic Review.

[19] Jones BF (2010). Age and great invention.. The Review of Economics and Statistics.

[20] Henrich J (2004). Demography and cultural evolution: How adaptive cultural processes can produce maladaptive losses - the Tasmanian case.. American Antiquity.

[21] Enquist M, Ghirlanda S, Jarrick A, Wachtmeister CA (2008). Why does human culture increase exponentially?. Theoretical Population Biology.

[22] Powell A, Shennan S, Thomas MG (2009). Late Pleistocene demography and the appearance of modern human behavior.. Science.

[23] Eriksson K, Enquist M, Ghirlanda S (2007). Critical points in current theory of conformist social learning.. Journal of Evolutionary Psychology.

[24] Enquist M, Ghirlanda S, Eriksson K (2011). Modelling the evolution and diversity of cumulative culture.. Philosophical Transactions of the Royal Society B.

[25] Sperber D (1996). Explaining culture: A naturalistic approach..

[26] Atran S (2001). The trouble with memes: Inference versus imitation in cultural creation.. Human Nature.

[27] Price GR (1970). Selection and covariance.. Nature.

[28] Basalla G (1988). The evolution of technology..

[29] Vincenti WG (1993). What engineers know and how they know it..

[30] Bentley RA, Hahn MW, Shennan SJ (2004). Random drift and culture change.. Proceedings of the Royal Society B.

[31] Mesoudi A, Lycett SJ (2009). Random copying, frequency-dependent copying and culture change.. Evolution and Human Behavior.

[32] Strimling P, Sjostrand J, Enquist M, Eriksson K (2009). Accumulation of independent cultural traits.. Theoretical Population Biology.

[33] Rendell L, Boyd R, Cownden D, Enquist M, Eriksson K (2010). Why copy others? Insights from the social learning strategies tournament.. Science.

[34] Boyd R, Richerson PJ, Nitecki M, Nitecki DV (1992). How microevolutionary processes give rise to history.. History and evolution.

[35] Cavalli-Sforza LL, Feldman MW (1981). Cultural transmission and evolution..

[36] Bloom P, Weisberg DS (2007). Childhood origins of adult resistance to science.. Science.

[37] Kelemen D (1999). The scope of teleological thinking in preschool children.. Cognition.

[38] Mesoudi A (2008). Foresight in cultural evolution.. Biology and Philosophy.

[39] Mesoudi A, O'Brien MJ (2008). The cultural transmission of Great Basin projectile point technology I: An experimental simulation.. American Antiquity.

[40] Mesoudi A (2008). An experimental simulation of the ‘copy-successful-individuals’ cultural learning strategy: Adaptive landscapes, producer-scrounger dynamics and informational access costs.. Evolution and Human Behavior.

[41] Sahlins M, Service E (1960). Evolution and culture..

[42] Lipo CP, O'Brien MJ, Collard M, Shennan S (2006). Mapping our ancestors: Phylogenetic approaches in anthropology and prehistory..

[43] Arthur WB (2009). The nature of technology..

[44] Ogburn WF (1950). Social change..

