# Supplementary material for: Variable Cultural Acquisition Costs Constrain Cumulative Cultural Evolution
Source: PLoS One. 2011 Mar 30;6(3):e18239. doi: 10.1371/journal.pone.0018239 (PMC3068160; doi:10.1371/journal.pone.0018239)
Supplement: Text S1 — Data sources for Figure 2A /Table S1. (DOC) [file pone.0018239.s001.doc]

**Supporting Information File S1 - Data sources for Figure 2A / Table S1**

The complete set of ages and historical dates plotted in Figure 2A is shown in Table S1. Educational data (the age at which children are taught and expected to know selected mathematical concepts) for ages 5-16 are taken from the English National Curriculum for Mathematics published in 1999 by the Department for Education and Employment, available here:

[http://curriculum.qcda.gov.uk/uploads/Mathematics%201999%20programme%20of%20study_tcm8-12059.pdf](http://curriculum.qcda.gov.uk/uploads/Mathematics 1999 programme of study_tcm8-12059.pdf)

Where age groups span multiple years, the mid-point of the period is taken (e.g. Key Stage 1, ages 5-7 is plotted as age 6). Educational data for ages 16-18 are taken from the Edexcel Mathematics A-Level specification, available here:

[http://www.edexcel.com/migrationdocuments/GCE%20New%20GCE/UA024850%20GCE%20in%20Mathematics%20issue%202%20180510.pdf](http://www.edexcel.com/migrationdocuments/GCE New GCE/UA024850 GCE in Mathematics issue 2 180510.pdf)

University level data (undergraduate and postgraduate taught masters level) are taken from Queen Mary University of London School of Mathematics website:

<http://www.maths.qmul.ac.uk/undergraduate/modules>

University level courses are assumed to proceed directly from school-leaving age with no break, so three-year undergraduate programmes are assumed to be taken from ages 18-21, and one-year masters courses at age 22. These ages are therefore conservative estimates, as many students will take study breaks in between these degrees. Furthermore, given that the United Kingdom has comparatively short undergraduate and postgraduate degree programmes, the equivalent ages for other countries may be significantly older, potentially increasing acquisition times further.

Historical data are taken from Wilder (1968) and Gittleman (1975). Given the ambiguity of historical sources, particularly from early civilizations such as the Babylonians where extensive written records do not exist, it is impossible to put exact dates on many of the early concepts. Further ambiguity arises when concepts have been discovered multiple times seemingly independently (e.g. discoveries in Ancient China that parallel subsequent independent discoveries in Europe, such as the formulation of Pascal’s triangle in China in 1100 and in Europe in 1653). Given that we are interested in cumulative cultural change, in these situations I have used dates of discoveries that formed part of a cumulative sequence. For example, where European scholars independently invented techniques formulated earlier by Chinese scholars, the later European date was used as Chinese mathematics did not contribute to subsequent mathematical developments. For later dates, I used the point at which an originator first published an eponymous law or theorem (e.g. Laplace's equation or Newton’s laws of mechanics), even though partial solutions may have existed before these publication dates.

**References**

Gittleman, A. (1975). *History of mathematics*. Columbus, OH: Merrill.

Wilder, R. L. (1968). *Evolution of mathematical concepts*. Milton Keynes: Open University Press.
