# Supplementary material for: Variable Cultural Acquisition Costs Constrain Cumulative Cultural Evolution
Source: PLoS One. 2011 Mar 30;6(3):e18239. doi: 10.1371/journal.pone.0018239 (PMC3068160; doi:10.1371/journal.pone.0018239)
Supplement: Text S2 — Derivation of Equation 5 . (DOC) [file pone.0018239.s002.doc]

**Supporting Information File S2 - Derivation of Equation 5**

No new traits can be learned by innovation when the cost of innovation *c_i_* exceeds the budget remaining after copying the previous generation’s set of *s* traits, given by (*λ* - *sc_s_*), i.e. when

(S1)

Rearranging this inequality gives the expression for the maximum number of traits that can be accumulated, *s_max_*, given in Eq. 5. Note that because *s* can only be an integer, the actual *s_max_* is the largest integer fulfilling Eq. 5 (i.e. the floor function ).
