# Supplementary material for: Variable Cultural Acquisition Costs Constrain Cumulative Cultural Evolution
Source: PLoS One. 2011 Mar 30;6(3):e18239. doi: 10.1371/journal.pone.0018239 (PMC3068160; doi:10.1371/journal.pone.0018239)
Supplement: Table S1 — Exact dates and ages plotted in Figure 2A . (DOC) [file pone.0018239.s003.doc]

**Table S1. Exact dates and ages plotted in Figure 2A.** Note that Figure 2A plots dates as years before present on a logarithmic scale, rather than in the BC/AD format used here. See Supporting Information File S1 for further details.

| **Concept (primary discoverer)** | **Year discovered** | **Age learned** |
| --- | --- | --- |
| Numerical notation (Babylonians) | 2000 BC | 6 |
| Geometry (Greeks) | 300 BC | 9 |
| Algebra (Al-Khwarizmi) | 825 | 12.5 |
| Trigonometry (Pitiscus) | 1595 | 15 |
| Logarithms (Napier) | 1614 | 17 |
| Calculus (Liebniz) | 1684 | 17 |
| Mechanics (Newton) | 1687 | 17 |
| Graph theory (Euler) | 1736 | 20 |
| Laplace's equation (Laplace) | 1825 | 20 |
| Group theory (Galois) | 1830 | 20 |
| Differential geometry (Riemann) | 1868 | 20 |
| Set theory (Cantor) | 1874 | 21 |
| Measure theory (Lebesgue) | 1902 | 22 |
| Relativity (Einstein) | 1905 | 21 |
